# Supplementary material for: Dynamic plasticity in phototransduction regulates seasonal changes in color perception
Source: Nat Commun. 2017 Sep 4;8:412. doi: 10.1038/s41467-017-00432-8 (PMC5583187; doi:10.1038/s41467-017-00432-8)
Supplement: Supplementary file 1 — Supplementary Information [file 41467_2017_432_MOESM1_ESM.pdf]

SI GUIDE

File Name: Supplementary Information
Description: Supplementary Figures, Supplementary Tables and Supplementary References.

File Name: Supplementary Data 1
Description: Differentially expressed genes in transition from SC to LW conditions. Black and blue characters indicate long day/warm temperature (LW)-up-regulated and -down-regulated genes, respectively. Genes highlighted in yellow are those involved in phototransduction.

File Name: Supplementary Movie 1
Description: Virtual medaka generated by three-dimensional computer graphics (3D-CG).

File Name: Peer Review File
Description:

Supplementary Figures

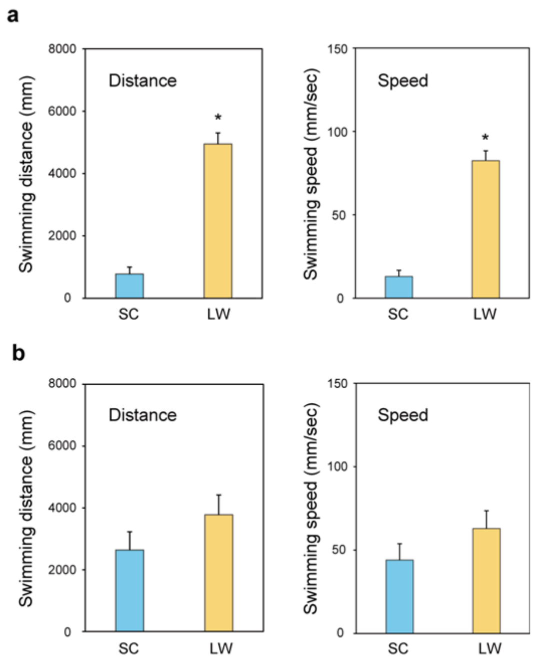

Supplementary Figure 1. Although activity level of short day/cool temperature (SC) fish is low, they have the capacity to move. (**a**) Significant differences were observed in the swimming distance (left; $P < 0.05$, *t*-test, mean + SEM, n = 10-12) and swimming speed (right; $P < 0.05$, *t*-test, mean + SEM, n = 10-12) between SC fish and long day/warm temperature (LW) fish in an open field tank. Swimming distance and speed were calculated from the data shown in Figure 1a. (**b**) No significant differences were observed in the swimming distance (left; $P > 0.05$, *t*-test, mean + SEM, n = 5-9) and swimming speed (right; $P > 0.05$, *t*-test, mean + SEM, n = 5-9) between SC and LW fish in a novel test tank. Swimming distance and speed were calculated from the data shown in Figure 1b.

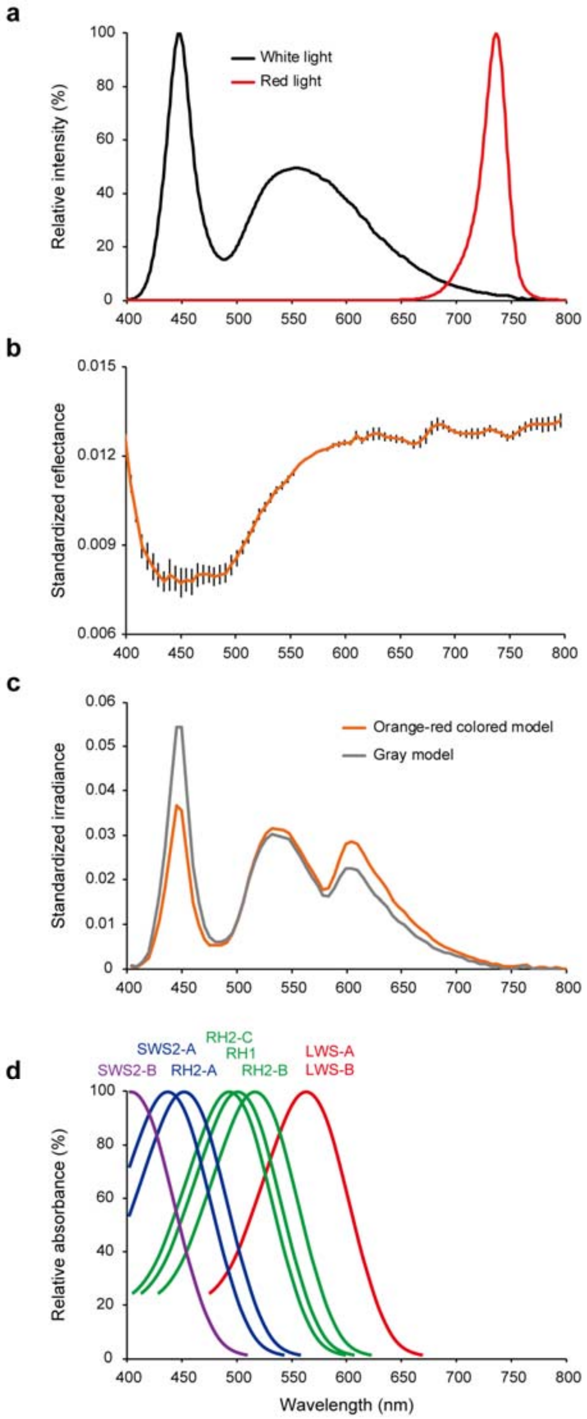

Supplementary Figure 2. Spectral curves for light sources, body color and visual pigments. (**a**) Relative radiant output of white and monochromatic red light. (**b**) Spectral reflectance from the caudal fin of medaka kept under long

day/warm temperature (LW) conditions (mean ± SEM, n = 4) (**c**) Spectral power distribution of orange-red colored model fish and gray model fish on the screen. Orange-red-colored model had high spectral power at long wavelengths covered by LWS. Note that gray model fish also contained spectral power in the red range. This is because gray is produced by mixing three-additive primary color beams (RGB). (**d**) Schematic drawing of absorption spectra of medaka photopigments. These absorption spectra were drawn by using a spectral template[1] according to the reported values for absorption maxima[2]. Note that although the λ_{max} of LWS is about 560 nm, LWS has been demonstrated to cover an unexpectedly wide range of wavelengths (up to 830 nm) under light-adapted conditions[3].

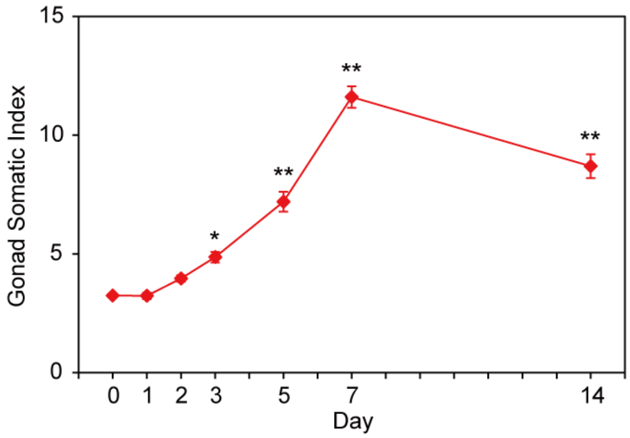

Supplementary Figure 3. Induction of gonadal development by LW. When fish kept under SC conditions were transferred to LW, a significant increase in the gonad somatic index (gonadal weight / body weight × 100) was first detected on day 3, and ovaries reached their full size on day 7 ($F_{6,35} = 73.8$, $P < 0.01$, ANOVA; $^{*}P < 0.05$, $^{**}P < 0.01$, vs. day 0, Dunnett's test; mean ± SEM, n = 6).

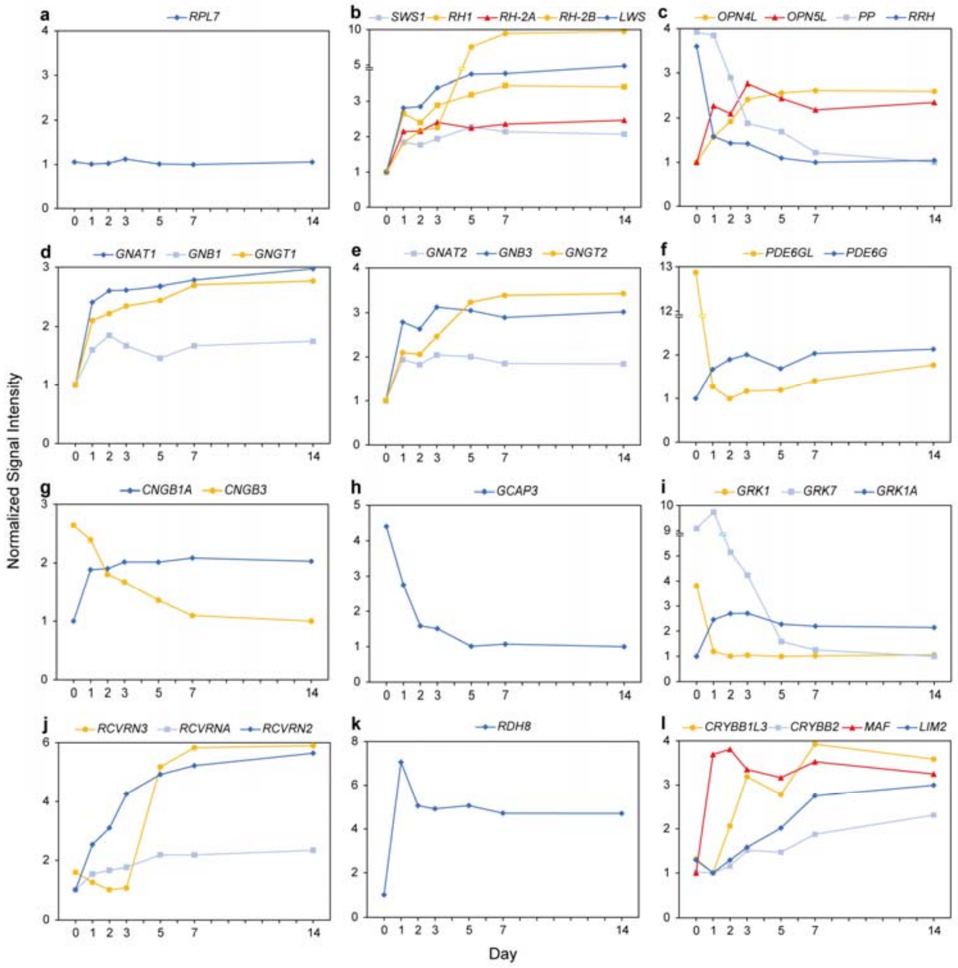

Supplementary Figure 4. Seasonal changes in the expression of genes involved in phototransduction determined by microarray analysis. (**a**) House-keeping gene. (**b**) Image-forming visual pigment genes. (**c**) Non-image-forming visual pigment genes. (**d**) Rod transducin genes. (**e**) Cone transducin genes. (**f**) Phosphodiesterase genes. (**g**) Cyclic-nucleotide-gated cation channel genes. (**h**) Guanylate-cyclase-activating protein gene. (**i**) Rhodopsin kinase genes. (**j**) Recoverin genes. (**k**) Retinol dehydrogenase gene. (**l**) Genes involved in lens formation. Data were normalized such that the minimum expression values over all time points for each gene is 1.

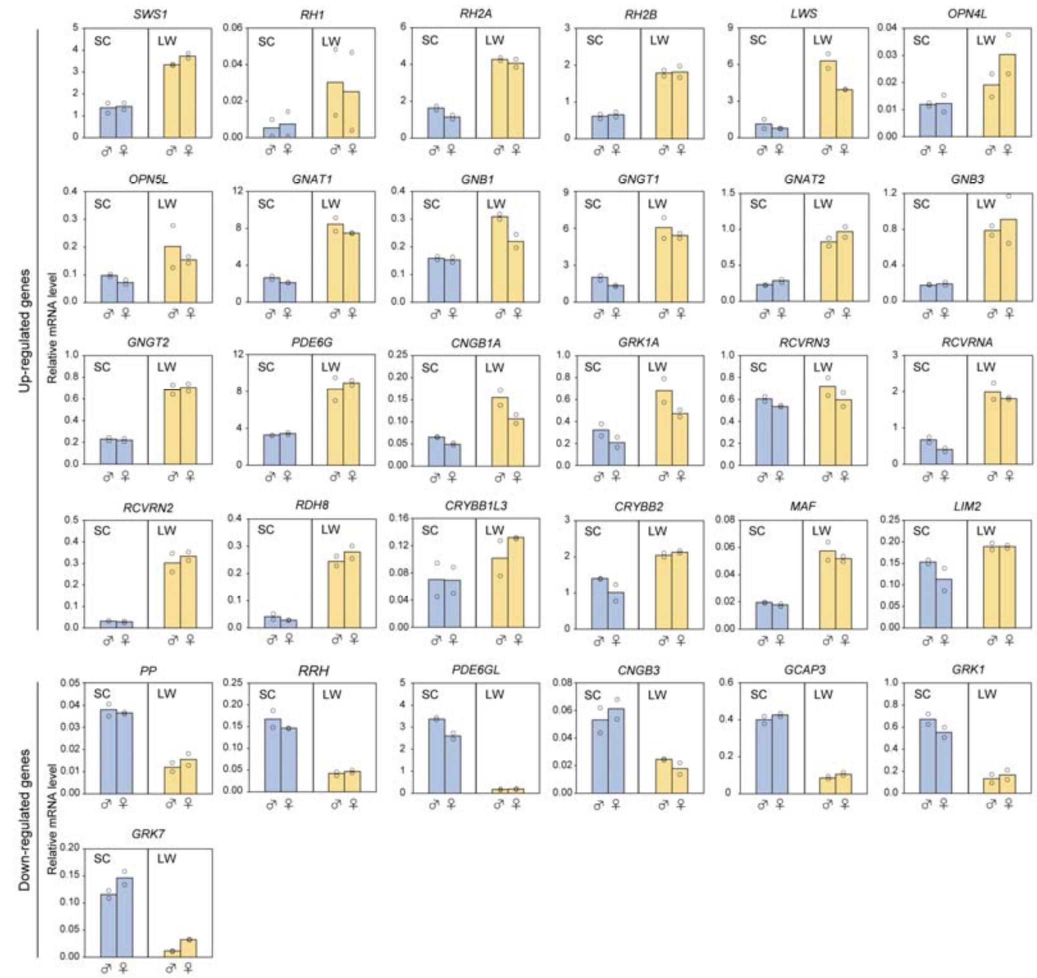

Supplementary Figure 5. Independent verification of microarray quantification in male and female medaka fish. Relative mRNA levels in the eye were measured by qPCR assay using medaka on days 0 (SC) and 14 after transfer from SC to LW (LW) conditions.

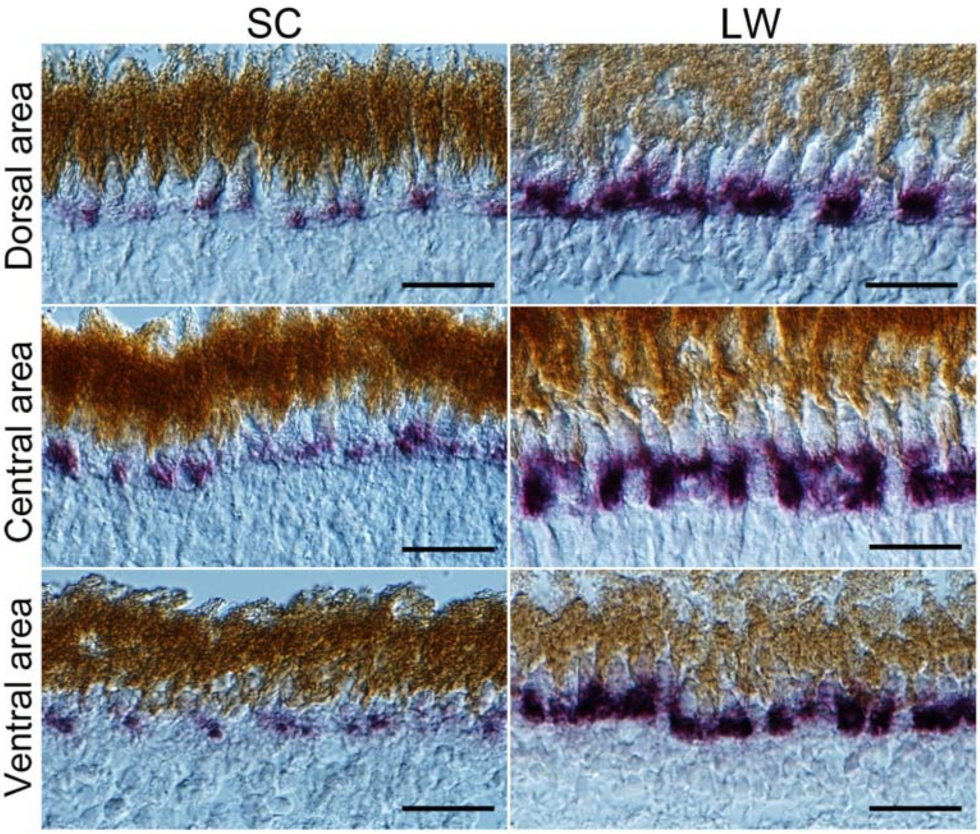

Supplementary Figure 6. Long day/warm temperature (LW)-induction of *LWS* is observed in whole retina. Representative high-resolution images from the dorsal, central and ventral areas of the retina taken by Differential Interference Contrast (DIC) microscope (Olympus BX63). Scale bars: 20 μm.

Supplementary Tables

Supplementary Table 1. Primer sequences used in the Q-PCR

Gene symbol	Primer sequence (sense)	Primer sequence (antisense)
RPL7	5' - aactggcctttgtcatcagg - 3'	5' - aaagttctgcagcttctccg - 3'
SWS1	5' - ggagtcggtacatccctgaa - 3'	5' - agttcagttgcgccagctat - 3'
RH1	5' - ttgaaggcttctttgccact - 3'	5' - aatgaccaacttccgctttg - 3'
RH-2A	5' - gagggattcatggcaacact - 3'	5' - ttcaaattcactgcaaccca - 3'
RH-2B	5' - gctggagtccttctgacctg - 3'	5' - tacactctggctcctggctt - 3'
LWS	5' - gggtagtcgtgtgcaaacct - 3'	5' - ttggtggaatcgtcttctcc - 3'
OPN4L	5' - accacatggacatcaagcaa - 3'	5' - acctggacacttcgttttgg - 3'
OPN5L	5' - gatcacgctgaaggcttttc - 3'	5' - cagcactctgcactcagagc - 3'
PP	5' - ggatgtttgtggtgtgcaag - 3'	5' - ctgtggtcccttacctggaa - 3'
RRH	5' - gcatcctttggtgaccctaa - 3'	5' - ctctttgccaagtcctccac - 3'
GNAT1	5' - ggagctagcgctgaagagaa - 3'	5' - ctggagaatcaggcaaaagc - 3'
GNB1	5' - cctcctgggtcatgacttgt - 3'	5' - aacctgaaaacacgtgaggg - 3'
GNGT1	5' - tcgacgacctcacagacaag - 3'	5' - ggaagaggacacccttgtga - 3'
GNAT2	5' - tgaagaagggccagaaagaa - 3'	5' - agatcgtgttcaacgctgtg - 3'
GNB3	5' - tgaagcaggacgtgattgag - 3'	5' - aaaaccggagacaattgtgc - 3'
GNGT2	5' - tgaagaagggccagaaagaa - 3'	5' - agatcgtgttcaacgctgtg - 3'
PDE6GL	5' - acgctgcacgtgtatctttg - 3'	5' - caactgtcagcgctgtgttt - 3'
PDE6G	5' - cctgctcttttctcgtttgc - 3'	5' - gaaacttacgcaagcaaggc - 3'
CNGB1A	5' - gctggcgagaactactaccg - 3'	5' - gaagtccagaaccggatcaa - 3'
CNGB3	5' - ttctgccgtttgacttgttg - 3'	5' - ttgagttcagtgatcgcctg - 3'
GCAP3	5' - cgtcaaaggagaaggtgagc - 3'	5' - tcatggaaatgctcaccaaa - 3'
GRK1	5' - gtcatgtgacgagctgagga - 3'	5' - aactgaatgcagggatcctg - 3'
GRK7	5' - acgagatggtggctggatac - 3'	5' - tctcaacgaggagccaaagt - 3'
GRK1A	5' - tatctcggatctgggtctgg - 3'	5' - ctaaaggttatgccgggaca - 3'
RCVRN3	5' - tgatggcactttggatttca - 3'	5' - aaaactggagtgggcctttt - 3'
RCVRNA	5' - aacatggggaacacgaagag - 3'	5' - caaatactcagaggccgagc - 3'
RCVRN2	5' - cgatgatgggactctggact - 3'	5' - aaccactcgcaaactcgagt - 3'
RDH8	5' - cagcaaagctgacaaaccaa - 3'	5' - agacatcgcagagcacacac - 3'
CRYBB1L3	5' - aactttcagggccgtaggat - 3'	5' - attattgtcgagtgcggacc - 3'
CRYBB2	5' - accagctgtaagggagagca - 3'	5' - acagccaggagcacaagatt - 3'
MAF	5' - cagactgccaaaaagcacaa - 3'	5' - ccaagagctgatcgtcatca - 3'
LIM2	5' - ctgtgctgttttgctggtgt - 3'	5' - gtcctgtcagcctttcttgc - 3'

Supplementary References

1. Govardovskii, V.I., Fyhrquist, N., Reuter, T., Kuzmin, D.G., Donner, K. In search of the visual pigment tempelate. *Vis. Neurosci*. **17**, 509-528 (2000).
2. Matsumoto, Y., Fukamachi, S., Mitani, H. & Kawamura, S. Functional characterization of visual opsin repertoire in medaka (*Oryzias latipes*). *Gene* **371**, 268-278 (2006).
3. Homma, N., Harada, Y., Uchiyama, T., Kamei, Y. & Fukamachi, S. Protanopia (Red color-blindness) in medaka: A simple system for making color-blind fish and testing their spectral sensitivity. *BMC Genet.* **18**, 10 (2017).
